# Immunotherapy for Early Stage Colorectal Cancer: A Glance into the Future

**DOI:** 10.3390/cancers12071990

**Published:** 2020-07-21

**Authors:** Romain Cohen, Qian Shi, Thierry André

**Affiliations:** 1 Department of Medical Oncology, Hôpital Saint-Antoine, Sorbonne Université, Assistance Publique-Hôpitaux de Paris (AP-HP), F-75012 Paris, France; thierry.andre@aphp.fr; 2 Department of Health Science Research, Division of Biomedical Statistics and Informatics, Mayo Clinic, Rochester, MN 55905, USA; shi.qian2@mayo.edu

**Keywords:** stage III, microsatellite instability, mismatch repair deficiency, oxaliplatin, immunoscore

## Abstract

Immune checkpoint inhibitors (ICI) have reshaped therapeutic strategies for cancer patients. The development of ICI for early stage colorectal cancer is accompanied by specific challenges: (i) the selection of patients who are likely to benefit from these treatments, i.e., patients with tumors harboring predictive factors of efficacy of ICI, such as microsatellite instability and/or mismatch repair deficiency (MSI/dMMR), or other potential parameters (increased T cell infiltration using Immunoscore^®^ or others, high tumor mutational burden, *POLE* mutation), (ii) the selection of patients at risk of disease recurrence (poor prognostic features), and (iii) the choice of an accurate clinical trial methodological framework. In this review, we will discuss the ins and outs of clinical research of ICI for early stage MSI/dMMR CC patients in adjuvant and neoadjuvant settings. We will then summarize data that might support the development of ICI in localized colorectal cancer beyond MSI/dMMR.

## 1. Introduction

Cancer immunotherapy is based on the idea that the host immune system can be stimulated to eliminate malignant cells. The discovery of immune checkpoints and immune checkpoint inhibitors (ICI) was a breakthrough in the history of medical oncology. Immunotherapy for colorectal cancer (CRC) began 40 years ago, with the development of levamisole for surgically treated localized CRC. This antihelmintic was found to display immunomodulatory effects and was evaluated in adjuvant setting for CRC patients in the 1980s [1,2]. Positive results reported by Mortel and colleagues with levamisole plus 5-fluorouracil paved the way for a decade of clinical trials testing various combinations of these two compounds, until the emergence of leucovorin plus 5-fluorouracil regimens [3,4,5]. Adding levamisole to this new standard failed to enhance outcomes, and this immunomodulatory agent disappeared from the therapeutic arsenal [6,7].

In the era of immune checkpoint blockade, CRC holds a singular position, with a minority of tumors being highly sensitive to ICI (i.e., CRC with microsatellite instability (MSI) and/or mismatch-repair deficiency (dMMR)), but a vast majority of cold CRC refractory to these compounds [8]. MSI is a molecular phenotype related to a deficient DNA MMR system, resulting from MMR gene germline mutations (i.e., Lynch syndrome) or from an epigenetic silencing of the MMR system (i.e., sporadic cancer), the latter being frequently associated with the *BRAF*^V600E^ mutation. Compared to MSS/pMMR (microsatellite stable/proficient MMR) CRC, MSI/dMMR tumors are also characterized by: poor differentiation, mucinous component, proximal location, female and older age (but also young patients for Lynch-related cases), distant lymph node metastases, and peritoneal carcinomatosis [9]. MSI/dMMR has long been used in adjuvant setting as a positive prognostic parameter and as a pre-screening test for Lynch syndrome. It is also used for therapeutic management since fluoropyrimidines alone are not indicated in the adjuvant setting for patients with stage II dMMR CRC, given their favorable survival and the lack of impact of chemotherapy in this situation [10,11]. In contrast, adjuvant therapy with a fluoropyrimidine alone is the standard of care for MSS/pMMR stage II CC harboring high-risk features. For stage III diseases, the association of a fluoropyrimidine and oxaliplatin for 3–6 months is recommended, whatever the MSI/dMMR status [12,13,14].

MMR deficiency is not, per se, a direct transforming event. Most genetic alterations found in dMMR tumors are somatic events that occur as a result of MSI. The MSI-driven oncogenic pathway leads to a high tumor mutational burden, with highly immunogenic neoantigens arising from frameshift mutations. As a consequence, MSI tumors are highly infiltrated by cytotoxic T lymphocytes [15,16,17,18]. Two signals are required to initiate an adaptive immune response by T cells: MHC-antigen peptide recognition by the T cell receptor and co-stimulation via an array of receptors interacting with cognate ligands on antigen-presenting cells. It has been shown that MSI tumors are likely to persist in their hostile microenvironment because of immunoescape and the dramatic overexpression of immune checkpoints [15]. Based on these findings, ICI have been developed in MSI/dMMR tumors.

While outcomes with ICI-treated CRC patients were initially disappointing in basket trials, results from phase II biomarker-guided, non-randomized trials were, in contrast, highly impressive for MSI/dMMR metastatic CRC [19,20,21,22,23]. Compared to standard of care chemotherapy ± bevacizumab or cetuximab, pembrolizumab as first-line therapy for patients with MSI/dMMR metastatic CRC is associated with a clinically meaningful and statistically significant improvement in progression-free survival, and should consequently become the new standard of care for these patients [24].

MSI/dMMR has now become a major predictor for the efficacy of ICI. Given its frequency in localized colon cancer (CC) (10–15% compared to 5% of metastatic CRC), the development of ICI in adjuvant setting may concern a sizable group of patients [25,26]. Moreover, results of the NICHE trial that showed impressive results with preoperative nivolumab plus ipilimumab for early stage MSI/dMMR CC but also for MSS/pMMR CC, has generated considerable attention for the implementation of neoadjuvant immunotherapy in CC [27].

In this review, we will focus on the key issues at stake in the development of ICI for patients with resected CC, with a particular interest for the MSI/dMMR population. Finally, we will highlight potential strategies to expand the use of ICI in localized CRC beyond the MSI/dMMR phenotype.

## 2. Immunotherapy as Adjuvant Treatment for Patients with Localized MSI/dMMR CC

### 2.1. What Is the Best Way to Screen Patients for MSI/dMMR? Immunochemistry, PCR or NGS?

At this time, there is no clear consensus concerning the way to screen patients for MSI/dMMR phenotype among agencies responsible of drug labelling (e.g., Food and Drug Administration, the European Medicines Agency). Considering MSI/dMMR as inclusion criterion for clinical trial enrollment, it is worthy to note that the frequency of MSI/dMMR misdiagnosis is not rare in real-life practice routine [28]. Therefore, attention should be devoted in MSI/dMMR diagnostic methods with potentially a systematic central review [29,30,31]. Two standard reference methods, namely immunohistochemistry (IHC) and polymerase chain reaction (PCR), are recommended for the detection of MSI/dMMR status. These methods are equally valid as the initial screening test for MSI/dMMR in CRC. Importantly, in contrast to MSI/dMMR testing with PCR, IHC can help identifying the affected gene and therefore directing germline mutation analysis. Furthermore, diagnostic performances of IHC are less affected by poor cellularity than PCR, which might be an issue with mucinous tumors or biopsies [32].

Next-generation sequencing (NGS) represents an alternative molecular test for the detection of tumor MSI/dMMR status and includes several techniques and algorithms [33,34,35,36,37,38]. It also enables the determination of the tumor mutational load. NGS has been reported to exhibit high concordance rates with both PCR and IHC, and two algorithms (namely MSISensor and FoudationOne Cdx1) have been approved by the US Food and Drug Administration. Nonetheless, the diagnostic accuracy of NGS-based algorithms is not as certain as these of IHC and PCR. Moreover, the applicability of NGS approaches in a real-life routine has not been demonstrated, and they should only be carried out at selected specialist centres or through validated central laboratory methods [30].

All in all, the most relevant approach for MSI/dMMR testing in the context of clinical trial enrollment of localized CRC patients seems to be an upfront testing with IHC using a four-antibody panel (immunostaining of MLH1, PMS2, MSH2, and MSH6 proteins) followed by molecular approaches (PCR) when IHC is equivocal [30,31,32].

### 2.2. Adjuvant Treatment and Prognostication of Patients With Localized MSI/dMMR CC

MSI/dMMR is a favorable prognostic feature in localized (stage I–III) CCs. Consequently, adjuvant therapeutic strategies are at risk to overtreat many patients with early stage MSI/dMMR CC, the majority of these patients being cured by surgery alone. Current guidelines recommend not to treat patients with stage II MSI/dMMR CC and to prescribe a combination of fluoropyrimidine and oxaliplatin for 3–6 months for stage III MSI/dMMR CC patients [26,39]. Moreover, 90% of patients with stage II MSI/dMMR CC are cured by surgery alone [40]. In a stage III setting, approximately 60% of the MSI/dMMR population is cured after surgical resection of the primary tumor, and 10–15% at most benefit from oxaliplatin-based adjuvant treatment [26,41]. All in all, almost one third of stage III MSI/dMMR CC patients will experience tumor relapse, but with an important prognostic heterogeneity depending on patient and disease characteristics [42].

Improving the prognostication of MSI/dMMR cancer patients is urgently needed to identify patients who are at risk of disease recurrence and to develop specific immunotherapeutic and/or immunochemotherapeutic strategies for this population. T4 stage and N2 stage (i.e., the high-risk stage III MSI/dMMR population) are currently the best-known prognosticators for the MSI/dMMR population [43,44]. In this population, three-year disease-free survival rates are approximately 60–65% for T4 and/or N2 stage III tumors, compared to 90% for low-risk MSI/dMMR stage III CC patients [44]. In other words, there is an urgent need for therapeutic improvements for patients with high-risk stage III MSI/dMMR CC, compared with patients with stage II and low-risk stage III MSI/dMMR CC whose prognosis is more favorable. Therefore, the most relevant population to target in the first place, with randomized trials testing adjuvant ICI, should be patients with T4 and/or N2 MSI/dMMR CC.

It is noteworthy that circulating tumor DNA might be a useful tool to select patients with early stage MSI/dMMR CC at risk of relapse (i.e., to avoid overtreatment in an adjuvant setting). Nonetheless, there are currently no data about circulating tumor DNA for localized MSI/dMMR CC.

### 2.3. Which ICI(s) in Adjuvant Setting for Localized MSI/dMMR CC?

The current standard of care for stage III MSI/dMMR CC patients is 3–6 months of fluoropyrimidine plus oxaliplatin [13], which is beneficial for one fifth of this population at the most. While overall survival in IDEA failed to statistically reject null hypothesis of non-inferiority in overall population, the 0.4% difference in five-year overall survival should be placed in clinical context, with the fact that non inferiority was consistently observed for CAPOX but not for FOLFOX [12]. For this reason, three months of CAPOX could be considered for all stage III MSI/dMMR CC, despite the fact no data concerning MSI/dMMR status is available in IDEA. This shortened duration of treatment is justified also by the fact that fluoropyrimidine alone seems not to work in early stage CC and by the fact that oxaliplatin is stopped before the end for the majority of patients for whom six months of therapy are planned.

However, one should keep in mind that the added value of adjuvant chemotherapy might be faded by the efficacy of ICI in the MSI/dMMR population. Therefore, the evaluation of ICI alone or in combination with chemotherapy are both valid options for MSI/dMMR CC patients that could be chosen depending on the prognosis of the targeted population (ICI versus chemotherapy for trials in low-risk patients, chemotherapy +/− ICI for trials targeting high-risk patients). There are currently two phase III randomized trials for the MSI/dMMR stage III CC population that test immune-chemotherapeutic strategies: the ATOMIC trial (FOLFOX +/− atezolizumab), and the POLEM trial (24 weeks of single agent fluoropyrimidine chemotherapy or 12 weeks of doublet, oxaliplatin-based chemotherapy +/− avelumab) [Table 1] [45]. A randomized double-blind phase II study of adjuvant pembrolizumab or placebo is ongoing for patients with MSI/dMMR solid tumors with persistent circulating tumor DNA following surgery is ongoing (NCT03832569).

Combinations of anti-PD1 and anti-CTLA4 monoclonal antibodies (e.g., nivolumab plus ipilimumab) seem more effective than anti-PD1 or anti-PDL1 agents alone (e.g., nivolumab, pembrolizumab, dostarlimab; durvalumab, avelumab) in terms of objective response rates that rise from 30% to 60% and one-year overall survival rate with a 15% improvement in a non-randomized phase II study [23]. Nonetheless there is currently no data from randomized trials comparing monotherapy to combination therapy. The ongoing CA209-8HW will provide useful data for the comparison of nivolumab with or without ipilimumab; NCT04008030) [46]. Moreover, even if there might be a significant added value of ICI combinations, they are associated with more frequent immune-mediated adverse events, which might be clinically less acceptable in adjuvant setting, where a majority of patients with MSI/dMMR CC are cured by surgery alone.

### 2.4. Biomarkers Predictive for ICI Efficacy among MSI/dMMR Cancer Patients

The exact mechanisms of de novo and acquired resistance to immunotherapy in MSI/dMMR CRC patients are not known but might be explained by biological diversity of the host immune system and the tumor biology. There is currently no predictive parameter able to clearly dichotomize ICI-resistant MSI/dMMR tumors from the others. Neither PD-1 expression, beta-2-microglobulin mutations or major histocompatibility complex class I expression, *BRAF*^V600E^ mutation nor Lynch syndrome status have not been associated with sensitivity to ICI [20,23,33]. Some authors have reported resistant cases in relation with intra-tumoral heterogeneity in MSI/dMMR status [47,48]. These rare cases (less than 1%) represent a real challenge for the diagnostic approach and therapeutic management [47,49].

The tumor mutational burden has been reported to be predictive of ICI efficacy among the MSI/dMMR mCRC population [50,51]. However, the sample sizes of these two works were small (*n* = 22 and *n* = 33).

The immune infiltrate might be another potential predictive biomarker [52]. It is known that the immune infiltrate may differ in quantity and quality within MSI/dMMR tumors, and 16–32% of MSI/dMMR CRC exhibit low levels of T cell infiltration [15,18,53]. The Immunoscore^®^ remains to be evaluated as a predictive factor for ICI efficacy among the MSI/dMMR population.

Ancillary translational studies are required to identify patients likely to benefit from ICI therapy among MSI/dMMR cancer patients.

### 2.5. Choosing the Most Accurate Statistical Methods

Although overall survival is the gold standard time-to-event end point in cancer clinical trials, surrogate end points have been developed in order to speed up therapeutic development and reduce clinical trial duration and costs. In adjuvant setting, disease-free survival is the most robust surrogate end point, highly correlated with overall survival for stage III CC patients, and therefore accepted by the Food and Drug Administration [54,55]. It is worthy to note that it has not been specifically validated for the MSI/dMMR population. Guidelines for the definition of time-to-events end points have been recently formulated by international experts: the consensual definition of disease-free survival includes all causes of death as an event as well as anastomotic and metastatic relapses, and second primary CRCs (non-colorectal primary tumors are not considered as an event) [56]. It is important since the MSI/dMMR population is strongly enriched with Lynch syndrome carriers who are at risk to develop second primary tumors. Though, the risk of second primary MSI/dMMR tumors among Lynch syndrome carriers might be reduced following ICI, too. Hence, considering a clinical trial of adjuvant treatment for MSI/dMMR CC patients, three-year disease-free survival seems a valid surrogate of overall survival.

Beyond the fact that there is an unmet need for treatment of patients with T4 and/or N2 stage III MSI/dMMR CC, targeting this high-risk population in randomized trials enables to reduce the number of patients to be included (potentially larger effect size), and consequently the duration of the trial (more events). However, it does not prevent investigators and statisticians to address the specificities of ICI effect on survival. The hazard ratio is largely used to quantify the treatment effect for time-to-event end points, but its use requires that there be proportional hazards in the treatment arms. However, non-proportional hazards have been frequently reported in ICI trials due to the long-term survival and delayed clinical effect [57,58]. Complementary methods to evaluate treatment effects such as the ratio of restricted mean survival time should be anticipated in pre-planned statistical analyses [59,60].

## 3. Moving to the Neoadjuvant Setting?

### 3.1. Lessons From the FOxTROT Study

Pre- or peri-operative therapeutic strategies have been successful in many gastro-intestinal cancer locations. The phase III FOxTROT study (NCT00647530) provided useful data about such strategies for CC patients [61]. In this randomized trial, 1052 patients with localized CC predicted as stage T3-4, N0-2, M0 by CT-scan were randomized to a perioperative sequence (6 weeks of FOLFOX, then surgery, then 18 weeks of FOLFOX) or the standard strategy (surgery then 24 weeks of FOLFOX). Neoadjuvant treatment was well tolerated and associated with evidence of histological regression in 59% of patients (4% of pathological complete response), histological downstaging and reduced rate of incomplete resections (5% vs. 10%). One should keep in mind that pathologic response has not been demonstrated as a surrogate marker of relapse-free survival or overall survival in CC. De facto, this study did not reached its primary endpoint, with no significant reduction of the two-year failure rate (14% vs. 18% in the control arm, hazard ratio = 0.77, *p* = 0.11).

The first issue raised by the FOxTROT study is the pre-operative evaluation of tumor stage and nodal stage (i.e., distinguishing stage II tumors from stage III ones). There is no consensual recommendation concerning baseline staging, notably the evaluation of nodal status using CT-scans of which diagnostic accuracy is poor [62]. In other words, there is a risk to over-stage stage I and stage II tumors and therefore over-treat patients with useless preoperative treatment, notably oxaliplatin, which is responsible for potentially long-term neuropathy and is not the standard of care for patients with stage II disease.

Investigators of the FOxTROT study reported important data about histological regression according to the MMR status. Intriguingly, rate of tumor regression after neoadjuvant FOLFOX was markedly reduced in MSI/dMMR tumors (*n* = 106) compared to the others (*n* = 592) [63]. No regression (tumor regression grade = 0) was observed in 73.6% of the MSI/dMMR tumors vs. 26.6% of the MSS/pMMR cases. The poor results for the MSI/dMMR group were unexpected given the efficacy of oxaliplatin plus fluoropyrimidine as an adjuvant treatment for this population [26,64], even if similar data have been reported for MSI/dMMR gastric cancer [65,66]. These results suggest that it might be irrelevant to combine ICI with chemotherapy in neoadjuvant setting for MSI/dMMR CC patients.

### 3.2. Moving Forward with the NICHE Study

In the NICHE trial, patients with early-stage CC received a neoadjuvant treatment with ipilimumab on day 1 plus nivolumab on day 1 and day 15 before surgery which was performed a maximum of six weeks after inclusion. Patients with pMMR tumors were randomly assigned to received celecoxib from day 1 until the day before surgery [27]. Forty patients were treated, with 21 dMMR and 20 pMMR tumors (on patient with both pMMR and a dMMR CC). Firstly, the treatment was well tolerated, and five patients (13%) who experienced grade 3–4 treatment-related toxicity. Notably, one patient experienced a grade 3 colitis two months after surgery, that required one dose of infliximab. This reinforces the necessity to carefully evaluate the benefit-risk balance of such neoadjuvant strategies for patients whose prognosis cannot nowadays be rigorously evaluated without the analysis of the surgical specimen, and who could have been cured by surgery alone.

Strikingly, all dMMR CC patients had a pathological response, with 95% of major pathological responses (≤ 10% of residual viable tumor in the surgical specimen), including 12 (60%) of complete pathological responses. With a median follow-up of 8.1 months, all dMMR CC patients from the NICHE study were alive and disease free. To note, similar histological results have been reported for patients with the resection of residual lesions after ICI treatment for metastatic MSI/dMMR CRC [67,68]. In the phase II VOLTAGE-A study evaluating nivolumab followed by radical surgery after chemoradiotherapy for patients with rectal cancer, 60% of complete pathological responses were observed among MSI/dMMR cases (three out of five) [69].

All in all, these impressive results for localized MSI/dMMR CRC patients highlight the neoadjuvant immunotherapy as a promising strategy that warrants further research. The reasoning underlying the strategy of neoadjuvant immunotherapy rests on its ability to induce T cell expansion, its greater utility at earlier stages of cancer when T cell function is less impaired, and its potential to reduce tumor size before surgery, possibly improving surgical outcomes [70]. As a matter of fact, results of the NICHE study might refine therapeutic strategies for early-stage CCs. One might suggest that therapeutic de-escalation with avoidance of surgical resection might be an option in the future. This perspective might be particularly relevant for patients with MSI/dMMR rectal cancer, for whom organ preservation is a clinically meaningful issue, even more since these tumors (5–10% of all rectal cancers at most) seem particularly sensitive to neoadjuvant chemoradiotherapy but resistant to induction systemic chemotherapy [71,72]. Nevertheless, this requires the development of technological tools to predict the occurrence of a complete pathological response following neoadjuvant immunotherapy.

## 4. Immunotherapy for Localized CC Beyond MSI/dMMR

### 4.1. The Other Lesson From the NICHE Study: Neoadjuvant Immunotherapy for MSS/pMMR Tumors

The NICHE study provided hypothesis-generating data for patients with MSS/pMMR tumors [27]. Four of 15 evaluable pMMR CC patients (27%) treated with nivolumab plus ipilimumab had a pathological response, with two complete pathological responses and one case harboring 1% of residual viable tumor. Four other patients had some evidence of a pathological response. Celecoxib did not seem to improve sensitivity to ICI treatment. Interestingly, responses in pMMR tumors were observed despite low TMB and low number of insertions-deletions (indels). This response rate among pMMR tumors is striking, even more so since the clinical activity of ICI for MSS/pMMR metastatic CRCs has been clearly disappointing [19]. These results confirm data observed in other cancer types, that early-stage cancers may be more responsive to ICI, especially as neoadjuvant treatment [73].

In this small cohort, the only biomarker found to predict response among pMMR tumors was the presence of T cells with co-expression of CD8 and PD1. Other potential factors such as CD3+ CD8+ FOXP3+ T cell infiltration, TMB, interferon-gamma score, tertiary lymphoid structures, TMB or the consensus molecular subtype (CMS) classification did not significantly differ between pMMR responders and non-responders. Nevertheless, the NICHE study clearly shows there is a window of opportunity for immune checkpoint inhibitors as neoadjuvant treatment for patients with early-stage MSS/pMMR CC.

### 4.2. Selecting Patients According to the Immune Microenvironment

The analysis of the microenvironment might be valuable for the selection of patients who might benefit from immunotherapeutic strategies. Currently, the only data concerning immunotherapy for early-stage CC originate from the NICHE study that detected a significant correlation between pathological response to neoadjuvant ICI and the CD8+ PD1+ T cell infiltrate in pMMR tumors.

Different methods of immune microenvironment evaluation have been developed for localized CC [17,74,75,76]. They provide important prognostic information: patients with low infiltration of CD3+ and CD8+ T cells exhibiting a higher risk of relapse, whatever the MSI/dMMR status [74]. The analysis of the Immunoscore^®^ in the IDEA France trial confirmed its strong prognostic value in stage III CC, independently of the MSI/dMMR status [77]. The three-year disease-free survival was 66.80% (95%CI 62.23–70.94) and 77.14% (95%CI 73.50–80.35) for patients with low or intermediate to high immunoscore, respectively. Moreover, a predictive value of the Immunoscore^®^ for disease-free survival benefit with a longer duration of FOLFOX (six months vs. three months) was detected for patients with an intermediate to high score. On the opposite, a lack of benefit from six-month FOLFOX was observed in patients with low immunoscore^®^.

These results show that a higher risk of relapse or death does not necessarily translate in a higher efficacy of adjuvant treatment. They highlight the Immunoscore^®^ as a potential useful tool to guide immuno(chemo)therapeutic strategies for early-stage CC, in both MSI/dMMR and MSS/pMMR populations. Nevertheless, except for the data about the CD8+ PD1+ T cell infiltrate on the 15 pMMR tumors from the NICHE study, there is currently no published data that may support the assumption that the evaluation of tumor-associated inflammatory microenvironment is predictive for the efficacy of ICI for patients with MSS/pMMR CC.

### 4.3. Targeting Tumors with High Tumor Mutation Load: Polymerase-Mutated CCs

Emerging literature shows that high TMB is predictive for the efficacy of ICI. In the context of CRC, the vast majority of hypermutated tumors are MSI/dMMR. In the TCGA CRC cohort (*n* = 276), 16% of samples (*n* = 44) exhibited a hypermutated phenotype (defined as a TMB greater than 12 mutations per 10^6^ bases). Among these, 37 were MSI. All MSS hypermutated tumors (*n* = 7) harbored exonucleasic proofreading domain *POLE*-mutations (DNA epsilon polymerase) [78]. Similarly, in a study by Stadler and colleagues, 31 out of 224 CRC samples were hypermutated, of which 3 were MSS and displayed a deleterious *POLE* mutation [79].

*POLE*-mutation CRC usually exhibits a MSS/pMMR phenotype, but are “ultra-mutated”, with a higher TMB than MSI/dMMR cancers [78,80,81]. These tumors are highly sensitive to immune checkpoint tumors [82,83,84,85]. They arise from the left colon and rectum, in younger males. Though, polymerase-mutated CRCs are infrequent (<1%) and associated with favorable outcomes, with rare metastatic relapse [86]. Therefore, the added value of ICI compared to surgery alone and/or surgery followed by conventional adjuvant chemotherapy might be hard to prove. It is noteworthy that patients with CRC harboring polymerase epsilon mutation are eligible for the POLEM trial [table].

All in all, the overlap between hypermutated CC and MSI/dMMR or *POLE*-mutated tumors seem complete. Given the technical pitfalls related to the assessment of TMB, the added value of this biomarker to MSI/dMMR and *POLE* mutation testing is uncertain. Still, since not all *POLE* mutations seem to be driving hypermutagenesis, with a hypermutated phenotype being restricted to specific hotspots, the evaluation of TMB might provide useful “functional” information [81,87].

## 5. Conclusions

Immune checkpoint blockade represents a major therapeutic breakthrough for cancer patients. The development of ICI in adjuvant situation, alone or in combination standard chemotherapy, for early CC faces several challenges.

For the MSI/dMMR population, the favorable prognosis associated with MSI/dMMR status in stage II and III CC, with few DFS events (relapse or death), raises questions about the feasibility of such studies, with the necessity of including a large number of patients to detect a small therapeutic effect. The good prognosis of early stage MSI/dMMR CC highlights the question of the benefit–risk balance of adjuvant therapies. Because of the high efficacy of ICI in MSI/dMMR CRC, one question concerns what should the experimental arm of a study be for this population of patients: ICI alone or combined with oxaliplatin-based chemotherapy? To conduct a study of adjuvant ICI therapy, with a reasonable number of patients and a chance to improve DFS, it is probably more feasible to focus on patients with T4 or N2 MSI/dMMR CC, for whom the expected magnitude of effect is high. Selecting patients with persistent circulating tumor DNA after surgery and a high risk of relapse might be a seductive strategy for which clinical studies are ongoing. Concerning MSI/dMMR rectal cancers, neoadjuvant strategies with ICI and/or chemoradiotherapy need to be evaluated in clinical trials.

For MSS tumors, new predictive biomarkers (tumor infiltrating lymphocytes, TMB or others) are required. In this context, the consideration of the immune microenvironment seems to be the next step to take, at least as a prognostic parameter, eventually as a therapy-guiding biomarker if its predictive value for ICI efficacy is demonstrated. Finally, one should keep in mind that designing biomarker-guided clinical trials is fraught with specific challenges that have to be addressed, especially in the context of immune checkpoint blockade.

## Figures and Tables

**Table 1:** Immunotherapy trials in adjuvant setting for CRC patients.

NCT Identifier	Trial Name	Phase	Condition	Intervention	Status
Non biomarker-guided trials					
NCT02415699	-	II-III	stage III	FOLFOX +/− DC-CIK *	not yet recruiting
NCT01890213	-	I	stage III	AVX701 vaccine after adjuvant therapy **	completed
NCT03904537	-	I-II	stage III	XELOX followed by an injection of anti-PD1 antibody-activated tumor infiltrating lymphocytes	recruiting
NCT03854799	AVANA	II	rectum; neoadjuvant	preoperative radiochemotherapy + avelumab (anti-PDL1)	recruiting
NCT03127007	R-IMMUNE	I-II	rectum; neoadjuvant	Preoperative radiochemotherapy + atezolizumab (anti-PDL1)	recruiting
NCT04123925	NICOLE	II	T3-4 CC; neoadjuvant	preoperative nivolumab (anti-PD1)	completed
Biomarker-guided trials					
NCT03026140	NICHE	II	neoadjuvant; MSI/dMMR (group 1)	nivolumab (anti-PD1) + ipilimumab (anti-CTLA4)	recruiting
NCT03926338	PICC	I-II	non metastatic CRC; dMMR	Toripalimab (anti-PD1) +/− celecoxib	recruiting
NCT02912559	ATOMIC	III	stage III; dMMR	FOLFOX+/− atezolizumab (anti-PDL1)	recruiting
NCT03827044	POLEM	III	stage III;dMMR or POLE mutant	fluoropyrimidine-based chemotherapy +/− avelumab (anti-PDL1)	recruiting
NCT03832569	-	II	resected R0 tumor with persistent ctDNA; dMMR	Pembrolizumab (anti-PD1) or placebo	recruiting
NCT04165772	-	II	advanced rectal cancer; dMMR	Dostarlimab (anti-PD1) followed by chemoradiotherapy and surgery	recruiting

*: autologous dendritic cells (DC) mixed with cytokine-induced killer; **: alphavirus replicon encoding the CEA protein.
